# Editorial: Quantum Structures in Cognitive and Social Science

**DOI:** 10.3389/fpsyg.2016.00577

**Published:** 2016-04-25

**Authors:** Diederik Aerts, Jan Broekaert, Liane Gabora, Sandro Sozzo

**Affiliations:** ^1^Center Leo Apostel for Interdisciplinary Studies, Free University of BrusselsBrussels, Belgium; ^2^Department of Psychology, University of British ColumbiaKelowna, BC, Canada; ^3^School of Management, Institute for Quantum Social and Cognitive Science, University of LeicesterLeicester, UK

**Keywords:** quantum structures, quantum cognition, decision theory, human cognition, cognitive modeling

A fundamental problem in cognitive and social science concerns the identification of the principles guiding human cognitive acts such as decision-making, categorization, and behavior under uncertainty. Identifying these mechanisms would have manifold implications for fields ranging from psychology to economics, finance, politics, computer science, and artificial intelligence. The predominant theoretical paradigm rests on a classical conception of logic and probability theory. According to this paradigm people make decisions by following the rules of Boole's logic, while the probabilistic aspects of these decisions can be formalized by Kolmogorov's probability theory. This classical approach was believed to provide a quite complete and accurate account of human decision-making at both a normative level (describing what people should do) and a descriptive level (describing what people actually do). However, starting from the seventies, experimental studies of conceptual categorization, human judgment and perception, and behavioral economics have revealed that this classical conception is fundamentally problematical, in the sense that the cognitive models based on these mathematical structures are not capable of capturing how humans make decisions in situations involving uncertainty. In the last decade, an alternative scientific paradigm has arisen that employs a different and more general modeling scheme; it uses the mathematical formalism of quantum theory to model situations and processes in cognitive and social science. This new approach has not only met with considerable success but is becoming increasingly accepted in the scientific community, having attracted interest from important scientists, top journals, funding institutions, and media. Prisoners' dilemmas, conjunction and disjunction fallacies, disjunction effects, violations of the Sure-Thing principle, Allais, Ellsberg and Machina paradoxes, are only some of the examples where the application of the quantum mechanical formalism has shown significant effectiveness over traditional modeling schemes of a classical type.

The Frontiers Research Topic “Quantum Structures in Cognitive and Social Science” present an overview of current research that applies the formalism of quantum theory to cognitive and socio-economic domains. The term “quantum” may be misleading. The aim here is not to investigate the microphysical processes occurring in the human brain and, as a consequence, driving human judgments. Rather, we inquire into the validity of quantum theory as a general, coherent, and unitary paradigm for human cognition. In this respect, this research benefits from studies into the axiomatic and operational foundations of quantum physics. The scope of this bold approach to human cognition is discovering general rules that associate the empirical phenomenology in these domains with states, measurements, and probabilities of outcomes in such a way that these entities are represented exactly as quantum theory in Hilbert space represents states, measurements, and probabilities of outcomes in the phenomenology of microphysics. The ensuing modeling is theory-based, not experiment-based; that is, the models are not built around a specific effect or experiment, although they are sometimes used in conjunction with empirical data to build a stronger case. The models are constructed following the general epistemological and technical constraints of quantum theory; hence the successes of this quantum theory-based modeling suggest that it might provide a general theory for human cognition.

This Research Topic develops around three main directions of research, as follows.

- The deep reasons underlying the success of the quantum paradigm in cognitive and socio-economic domains are investigated.
- Further empirical situations are identified in these domains where the quantum formalism presents advantages with respect to traditional modeling schemes, and new genuine quantum structures appear.
- The application of this quantum paradigm is extended to novel and barely explored domains.

The first set of results concern knowledge representation and conceptual categorization. Aerts et al. analyze the results of a cognitive test on conjunctions and negations of natural concepts, showing that a quantum-theoretic probabilistic model in Hilbert space faithfully represents the collected data, at variance with a set-theoretic Kolmogorovian model. This result is explained by assuming the existence of two types of reasoning in human cognition, a dominant emergent reasoning, and a secondary logical reasoning. Some mathematical aspects of this quantum-theoretic model on conceptual conjunctions and negations are developed in Veloz and Desjardins through the introduction of unitary operators in Hilbert space. Aerts et al. show instead that the quantum-theoretic approach Aerts et al. can be interpreted as a suitable generalization of Rosch's prototype theory, where prototypes are context-dependent and may interfere when concepts combine.

The second set of results concern the modeling of human decision-making. Moreira and Wichert explore an alternative quantum-theoretic approach, the quantum-like Bayesian network, to describe the paradoxes related to the violation of the Sure-Thing Principle in experiments on human judgments. Their model is in a good agreement with different sets of empirical data. The opinion paper in Pothos et al. reviews some current progress on the quantum similarity model in Hilbert space recently proposed by Pothos et al. which correctly represents human similarity judgments. Decision-making errors and preference reversal are also investigated in Yukalov and Sornette within their quantum decision theory. Wang and Busemeyer analyze the notion of complementarity in human cognition, and claim that the way in which it is used in quantum physics can also be helpful in cognitive science. Human perception is the object of the study in Khrennikov, where the author develops a quantum model of the sensation-perception dynamics, illustrating it by means of the model of bistable perception of a specific ambiguous figure, the Schröder stair. Finally, Tressoldi et al. identify a significant violation of temporal Bell inequalities in a set of cognitive tests. The violation indicates, according to the authors, the presence of temporal entanglement between binary human behavioral unconscious choices at a given time and binary random outcomes at a different time. In all these approaches, the presence of quantum structures in cognition is determined by the fact that the cognitive systems under investigation share a common feature, namely contextuality. A different position with respect to the presence of contextuality in cognition is assumed in Zhang and Dzhafarov, where the authors apply a theory of (non)contextuality to analyze series-parallel (SP) mental architectures.

The third set of results concern advanced applications of the quantum-mathematical formalism to wider ranges of social science. Bisconti et al. propose an inverse Potts model, typically used in statistical quantum field theory, to reconstruct the node states in a real-world social network. Haven explores the properties of two types of potential functions, inspired by classical and quantum physics that can be potentially employed to model financial information, including preferences toward risk and uncertainty. Finally, Dalla Chiara et al. investigate different, but mutually related, aspects of parallelism within the framework of quantum computation, cognition and music, and study potential applications of quantum computational semantics in both natural and musical language.

Leaving aside the specific differences between the approaches above, most of them agree in claiming that quantum structures are systematically present in cognitive and social science phenomena, and that quantum-inspired models are more efficient than traditional set-theoretic models of probability. Is “quantum” the end of the story? Is Hilbert space really the place where all these phenomena can be modeled? Is there any empirical deviation from quantum predictions? We do not have yet an answer to these questions. This is why we believe that the road that will lead further to possibly a generally accepted quantum theory of human decision-making will still be full of fascinating surprises.

## Author contributions

All authors listed, have made substantial, direct, and intellectual contribution to the work, and approved it for publication.

### Conflict of interest statement

The authors declare that the research was conducted in the absence of any commercial or financial relationships that could be construed as a potential conflict of interest.

